# Increased Sensitivity to Broadly Neutralizing Antibodies of End-Stage Disease R5 HIV-1 Correlates with Evolution in Env Glycosylation and Charge

**DOI:** 10.1371/journal.pone.0020135

**Published:** 2011-06-16

**Authors:** Marie Borggren, Johanna Repits, Jasminka Sterjovski, Hannes Uchtenhagen, Melissa J. Churchill, Anders Karlsson, Jan Albert, Adnane Achour, Paul R. Gorry, Eva Maria Fenyö, Marianne Jansson

**Affiliations:** 1 Department of Laboratory Medicine, Lund University, Lund, Sweden; 2 Macfarlane Burnet Institute for Medical Research and Public Health, Melbourne, Australia; 3 Department of Medicine, Monash University, Melbourne, Australia; 4 Center for Infectious Medicine (CIM), Department of Medicine, Karolinska Institutet, Stockholm, Sweden; 5 Department of Infectious Medicine, South Hospital, Stockholm, Sweden; 6 Department of Microbiology, Tumor and Cell Biology, Karolinska Institutet, Stockholm, Sweden; 7 Department of Microbiology and Immunology, University of Melbourne, Parkville, Australia; University of Toronto, Canada

## Abstract

**Background:**

Induction of broadly neutralizing antibodies, such as the monoclonal antibodies IgGb12, 2F5 and 2G12, is the objective of most antibody-based HIV-1 vaccine undertakings. However, despite the relative conserved nature of epitopes targeted by these antibodies, mechanisms underlying the sensitivity of circulating HIV-1 variants to broadly neutralizing antibodies are not fully understood. Here we have studied sensitivity to broadly neutralizing antibodies of HIV-1 variants that emerge during disease progression in relation to molecular alterations in the viral envelope glycoproteins (Env), using a panel of primary R5 HIV-1 isolates sequentially obtained before and after AIDS onset.

**Principal Findings:**

HIV-1 R5 isolates obtained at end-stage disease, after AIDS onset, were found to be more sensitive to neutralization by TriMab, an equimolar mix of the IgGb12, 2F5 and 2G12 antibodies, than R5 isolates from the chronic phase. The increased sensitivity correlated with low CD4^+^ T cell count at time of virus isolation and augmented viral infectivity. Subsequent sequence analysis of multiple *env* clones derived from the R5 HIV-1 isolates revealed that, concomitant with increased TriMab neutralization sensitivity, end-stage R5 variants displayed envelope glycoproteins (Envs) with reduced numbers of potential N-linked glycosylation sites (PNGS), in addition to increased positive surface charge. These molecular changes in Env also correlated to sensitivity to neutralization by the individual 2G12 monoclonal antibody (mAb). Furthermore, results from molecular modeling suggested that the PNGS lost at end-stage disease locate in the proximity to the 2G12 epitope.

**Conclusions:**

Our study suggests that R5 HIV-1 variants with increased sensitivity to broadly neutralizing antibodies, including the 2G12 mAb, may emerge in an opportunistic manner during severe immunodeficiency as a consequence of adaptive molecular Env changes, including loss of glycosylation and gain of positive charge.

## Introduction

The intra-host evolution of human immunodeficiency virus type 1 (HIV-1) is facilitated by an error-prone reverse transcriptase (RT) and a high viral turnover [1]. After transmission a population of distinct but closely related viruses is established and, in constant interplay with selective forces from the host immune system or therapeutic agents, the population evolves during the course of the infection [2]. The viral envelope glycoprotein gp120/gp41 complex (Env) has been shown to exhibit the greatest diversity among viral proteins [3].

In the course of the entry process HIV-1 binding via gp120 to CD4 on the cell surface initiates a series of events including binding of the coreceptors CCR5 and/or CXCR4 and, ultimately, gp41-mediated fusion of the viral and cell membranes [4]. CCR5-restricted (R5) viruses predominate in the early asymptomatic stages of HIV-1 infection [5]. Viruses able to use CXCR4 instead of, or in addition to CCR5, for cell entry (X4, or R5X4 viruses, respectively) may emerge later during the disease course and their appearance has been correlated to accelerated progression to AIDS [6]–[8]. However, most infected individuals progress to AIDS while maintaining an exclusive R5 virus population [7]–[10]. We and others have previously studied the evolution of phenotypic and molecular properties of R5 viruses in patients progressing to AIDS while maintaining isolates with an exclusive R5 phenotype [10]–[20]. In these studies we demonstrated that R5 viruses with increased fitness, altered receptor interactions and reduced sensitivity to inhibition by HIV-1 entry inhibitors [10]–[14], [17], [19] may emerge after onset of AIDS. We also described molecular alterations in the R5 Env, including increased net positive charge in gp120 along with disease progression [18].

Since Env is exposed at the viral surface it is also the target for neutralizing antibodies, which can be detected a few months after transmission [21], [22]. Transmission of the virus from one individual to another is a bottleneck for virus diversity and the transmitted viruses have been reported to be relatively sensitive to neutralization [23], [24]. Following development of HIV-1, specific antibody escape variants will rapidly be selected resulting in enhanced diversity and a more neutralization-resistant population [21], [25]. However, many primary isolates can still be neutralized by a few broadly neutralizing antibodies including IgGb12 [26]–[28], 2F5 [29]–[31] and 2G12 [29], [32]–[35]. IgG1b12 recognizes an epitope that overlaps with the CD4 binding site on gp120 [28], 2F5 binds to a conserved linear epitope within the membrane proximal external region (MPER) of gp41 [30], [36] and 2G12 recognizes specific oligomannose glycans on the outer face of gp120 [35], [37], [38]. The HIV-1 Env is heavily glycosylated and Env glycosylation has been suggested to be part of a viral immune escape strategy [25], [39]. Previous studies have also suggested an enlargement of the Env glycan shield during the immunocompetent phase of the HIV-1 disease [24], [40], [41].

Despite the relative conserved nature of epitopes targeted by broadly neutralizing antibodies, mechanisms underlying the sensitivity of circulating HIV-1 variants to these antibodies are not fully understood. In this study we have analyzed virus sensitivity to broadly neutralizing antibodies in relation to Env modifications, including changes in glycosylation and charge, of HIV-1 R5 variants evolving during end-stage disease progression. By the use of a unique panel of R5 isolates obtained sequentially before and after AIDS onset at severe immunodeficiency we here reveal that end-stage R5 viruses display increased sensitivity to neutralization by the TriMab mix of broadly neutralizing monoclonal antibodies (MAbs) IgGb12, 2F5 and 2G12. Furthermore, we show that increased sensitivity to TriMAb neutralization correlates with a sharp decline in CD4^+^ T cell count, increase in viral infectivity and Env with molecular alterations including reduced numbers of potential N-linked glycosylation sites (PNGS) and enhanced positive charge. Virus sensitivity to neutralization by the individual 2G12 MAb was also found to correlate with viral infectivity and numbers of PNGS and positive charge of Env.

## Results

### End-stage R5 HIV-1 display increased sensitivity to broadly neutralizing monoclonal antibodies

**Figure 1:**
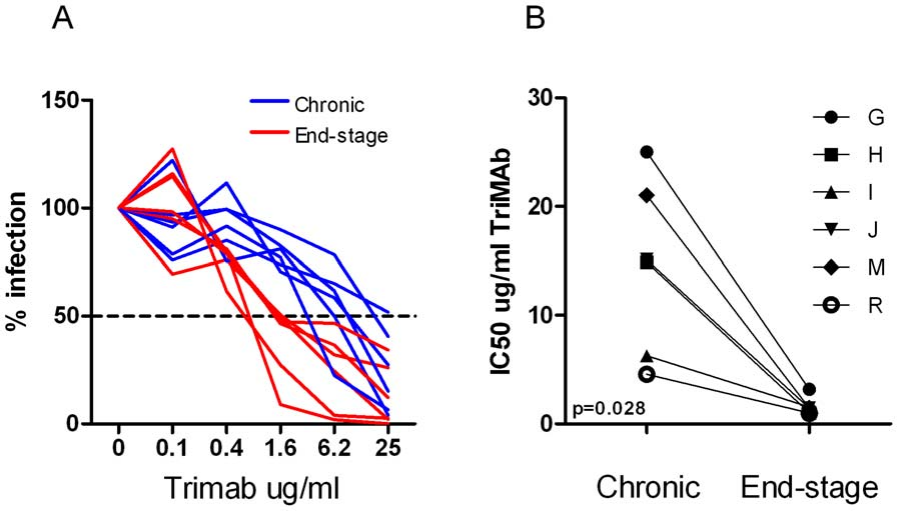
Sensitivity of sequential chronic and end-stage R5 viruses to neutralization by TriMAb. a) Percent TriMAb neutralization of chronic stage R5 isolates (blue lines) and end-stage R5 isolates (red lines), b) TriMAb IC50 of chronic and end-stage R5 isolates.

**Figure 2:**
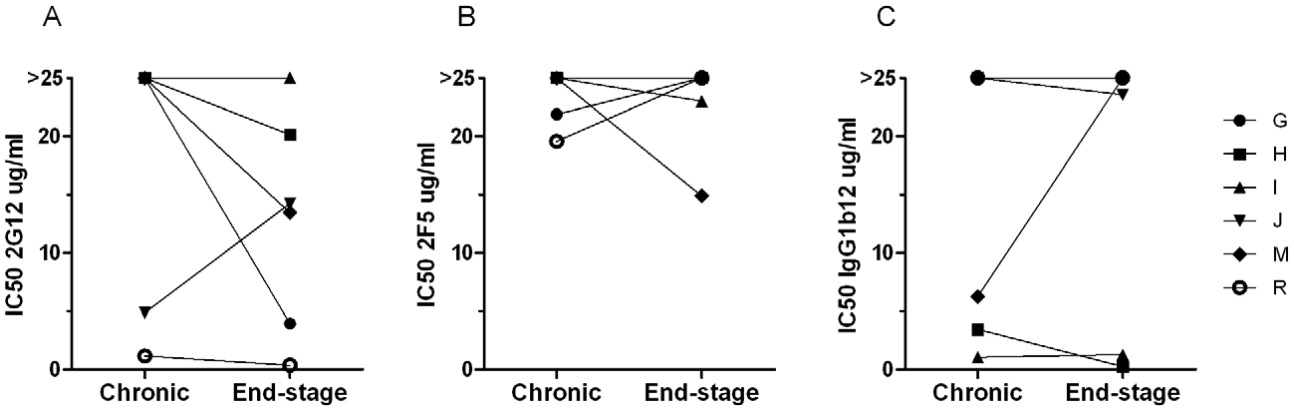
Sensitivity of sequential R5 viruses to neutralization by the 2G12, 2F5 or IgG1b12 monoclonal antibodies. Depicted are a) 2G12, b) 2F5 and c) IgG1b12 IC50 results from the analysis of neutralization sensitivity of sequential chronic and end-stage R5 viruses tested with each individual monoclonal antibody.

In order to explore whether HIV-1 R5 variants evolving during end-stage disease display altered sensitivity to broadly neutralizing antibodies, we set out to analyze virus neutralization sensitivity using a mix of the well characterized and broadly neutralizing human MAbs 2F5, 2G12 and IgG1b12, known as TriMAb. Sequentially obtained chronic and end-stage primary R5 isolates (Table 1) were tested in parallel against TriMab, using a plaque reduction assay with U87.CD4-CCR5 cells as target cells. R5 isolates from end-stage disease were found to be more sensitive to neutralization by the TriMAb mix than the corresponding R5 viruses from the chronic phase (Figure 1a). Accordingly, end-stage AIDS R5 virus from all patients displayed reduced TriMAb IC50 (p = 0.028, Figure 1b) in the U87-based neutralization assay. When TriMAb IC90 was analyzed end-stage R5 virus of three patients displayed enhanced sensitivity as compared to corresponding chronic stage R5 virus, while both chronic and end-stage R5 virus of three other patients was not neutralized to 90% at the highest concentration tested (data not shown). In agreement with the previously published concordance between the U87-based neutralization assay and the conventional PBMC-based neutralization assay [42], [43], our results on increased TriMab neutralization sensitivity of end-stage R5 viruses were confirmed when tested in a PBMC-based neutralization assay (data not shown). Furthermore, increased sensitivity to TriMAb neutralization correlated with reduced CD4^+^ T cell count at time of R5 virus isolation (p<0.001, r = 0.84, Table 2). We next tested chronic and end-stage R5 isolates for sensitivity to the individual 2F5, 2G12 and IgG1b12 MAbs. Several of the patients exhibited R5 virus from both chronic and end-stage disease that were not neutralized to 50% by the individual Mabs, even though all viruses could be neutralized to 50% using the same concentration of TriMAb. Thus, none of the MAbs could alone significantly distinguish neutralization sensitivity of virus from chronic and end-stage disease (Figure 2a–c). Still, end-stage R5 viruses tended to be more sensitive to 2G12 neutralization since five out of six end-stage R5 viruses were neutralized, in contrast to only two out of six chronic stage R5 viruses (Figure 2a). Taken together, these findings suggest that R5 HIV-1 variants with increased sensitivity to broadly neutralizing antibodies may emerge during severe immunodeficiency.

**Table 1:** Patient clinical status, CD4 count and virus coreceptor use.

Patient^a^	Isolate	CD4count^b^	Monthsto AIDS^c^	Clinical status	Coreceptoruse^d^
G	1228	260	-9	Chronic Asympt.	CCR5
	4481	5	+26	End-stage AIDS	CCR5
H	624	290	−27	Chronic Asympt.	CCR5
	3899	6	+6	End-stage AIDS	CCR5
I	5013	140	−30	Chronic Asympt.	CCR5
	8616	90	+11	End-stage AIDS	CCR5
J	1372	220	−11	Chronic Asympt.	CCR5
	5714	20	+20	End-stage AIDS	CCR5
M	668	750	−54	Chronic Asympt.	CCR5
	7363	20	+20	End-stage AIDS	CCR5
R	6322	200	−2	Chronic Asympt.	CCR3+CCR5
	8004	9	+16	End-stage AIDS	CCR3+CCR5

a Patient code according to [10]

b CD4^+^ T cells/µl blood at time of virus isolation.

c Time point of virus isolation related to months before and after AIDS diagnosis.

d Coreceptor use determined by infection of U87.CD4 and GHOST(3) coreceptor indicator cell lines expressing CCR2b, CCR3, CCR5, CXCR4, CXCR6 or BOB [10].

**Table 2:** Correlations between TriMAb sensitivity, Env PNGS, Env net charge, virus infectivity and patients CD4+ T cell count.

	TriMAb IC50^a^	PNGS in gp160^b^
**PNGS in gp160** b	p = 0.002, R = 0.80^e^	-
**gp160 net charge** b	p = 0.004, R = −0.76	p = 0.029, R = −0.63
**Virus infectivity** c	p = 0.042, R = −0.65	p = 0.014, R = −0.74
**CD4+ T cell count** d	p<0.001, R = 0.84	p = 0.019, R = 0.66

a TriMAb sensitivity of R5 isolates, assessed as IC50 µg/ml.

b Numbers of PNGS and net positive charges are the average numbers of four Env sequences per R5 isolate

c Virus infectivity evaluated as plaque forming units in U87.CD4-CCR5 cells.

d Patient CD4^+^ T-cell count at time of R5 virus isolation.

### Env of end-stage HIV-1 R5 virus variants display reduced glycosylation and increased positive charge concomitant with increased sensitivity to TriMab

**Figure 3:**
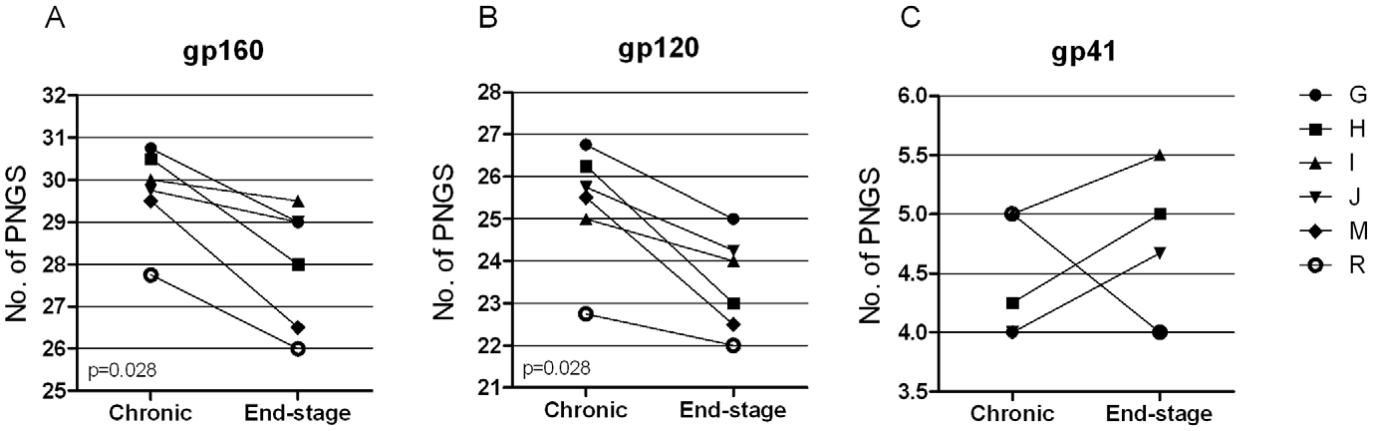
Evolution of PNGS modifications in HIV-1 R5 Env during end-stage disease progression. Differences in numbers of PNGS within a) gp160, b) gp120 and c) gp41 comparing the average PNGS numbers of four R5 sequences per isolate obtained longitudinally at the asymptomatic chronic phase and after AIDS onset at end-stage disease.

**Figure 4:**
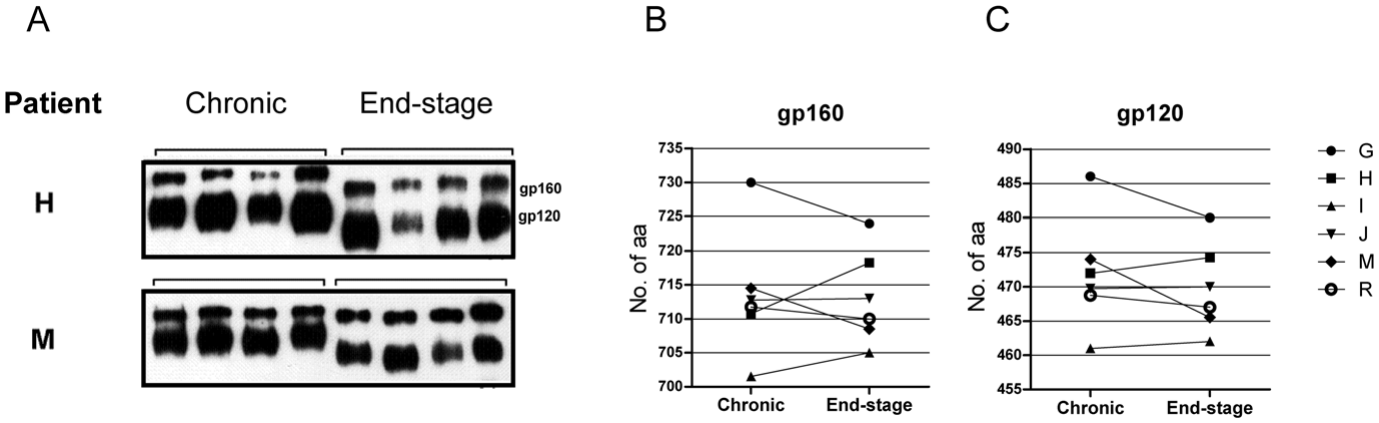
Molecular weight and peptide backbone length of Env from sequential HIV-1 R5 viruses. a) Mobility in SDS-PAGE gel of gp120 and gp160 clones from R5 HIV-1 isolates as assessed by Western Blot. The results of four Env clones for each R5 isolate obtained during chronic and end-stage disease from patients H and M are illustrated. Differences in amino acids sequence length of b) gp160 and c) gp120 comparing the average amino acids length of four R5 Env sequences per isolate obtained longitudinally at the chronic phase and at end-stage disease.

To examine whether emergence of R5 HIV-1 with increased sensitivity to broadly neutralizing antibodies was paralleled by Env evolution during end-stage disease, we analyzed amino acid modifications that could lead to altered glycosylation pattern. Numbers of PNGS in 48 *env* clones derived from the sequentially obtained primary R5 isolates were analyzed. The average number of PNGS for each isolate was calculated from the sequences of four different clones. A significant reduction in numbers of PNGS within gp160, as well as gp120, was observed when comparing R5 viruses isolated at end-stage disease with those from the chronic phase (p = 0.028 in both cases; Figure 3a and 3b), while no such clear pattern was apparent in gp41 (Figures 3c). We also noted that mutations leading to loss of PNGS mainly clustered in the gp120 variable regions, and in particular in the V2 and V4 regions (Figure S1). Conversely, the number of PNGS in the V3 loop was conserved between the two time points (Figure S1). Loss of glycosylation in end-stage Env sequences was further supported by western blot analysis. Here we noted that the molecular weight of gp160 and gp120 clones from the end-stage R5 isolates were lower when compared to clones from corresponding earlier isolate (Figure 4a). Additionally, end-stage Env clones that were de-glycosylated following treatment with PNGaseF displayed similar motility as compared to corresponding PNGaseF treated clones from the chronic stage (data not shown). Furthermore, length of the gp160 and gp120 amino acid sequences did not appear to differ in a consistent manner between chronic and end-stage R5 Env clones (Figure 4b and 4c). Reduced numbers of PNGS in gp160 correlated instead with decreasing CD4 counts at time of R5 virus isolation (Table 2). Since we previously reported on the development of R5 virus variants displaying Env with increased net positive charge in parallel with increased infectivity [17], [18], we analyzed PNGS numbers in relation to Env net positive charge and viral infectivity. We here found that reduced PNGS numbers in Env correlated with increased viral infectivity, assessed as plaque forming units in U87.CD4-CCR5 cultures, and Env with increased net positive charge (Table 2). To analyze whether evolution in R5 virus sensitivity to broadly neutralizing antibodies was associated with the observed Env modifications, we next assessed virus sensitivity to TriMAb in relation to numbers of PNGS and net charge of Env. We found that reduced TriMab IC50 correlated with reduced numbers of PNGS and increased net positive charge in Env (Table 2). In relation to viral fitness, we observed that R5 variants with increased sensitivity to TriMAb also were more infectious in the U87.CD4-CCR5 cultures (Table 2). Hence, we conclude that R5 HIV-1 variants with increased sensitivity to TriMAb neutralization may emerge during severe immunodeficiency and display augmented infectivity, in addition to Env with reduced glycosylation and an increase in net positive charge.

### R5 isolates with increased 2G12 sensitivity appear at low CD4^+^ T-cell count and display Env modifications

**Figure 5:**
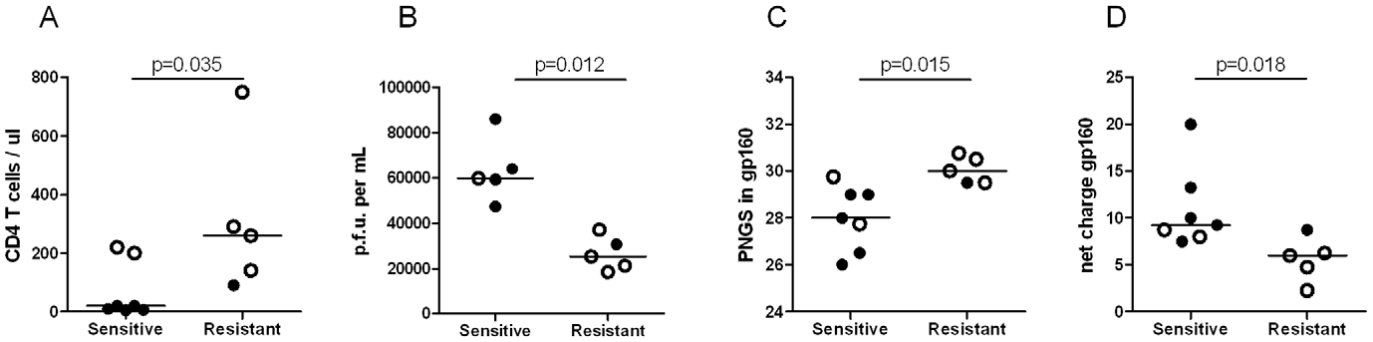
Patient status, viral infectivity and Env characteristics of 2G12 sensitive and resistant R5 isolates. Difference in a) CD4^+^ T cell count at time of virus isolation, b) viral infectivity, evaluated as plaque forming units in U87.CD4-CCR5 cells, c) gp160 PNGS numbers and d) gp160 net positive charge of R5 virus isolates being either sensitive (IC50 ≤25 µg/ml) or resistant (IC50 >25 µg/ml) to 2G12 neutralization. Presented PNGS numbers and net positive charge represent the average of four Env sequences per R5 isolates. Open circles represent chronic stage viruses, filled circles represent end-stage viruses.

**Figure 6:**
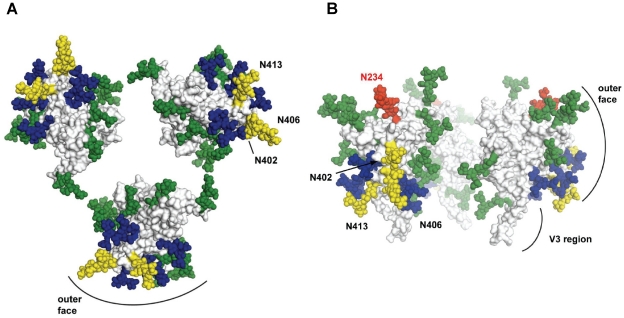
Models on gp120 localization of lost PNGS in end-stage virus in relation to 2G12 epitope. a) Surface representation of a molecular model of trimeric gp120 including glycosylation at PNGS, represented as spheres. The trimer is depicted from the orientation of the target cell with the V3 region facing the viewer. The PNGS that define the core epitope of 2G12 as well as the changes in PNGS from chronic to end-stage R5 viruses (in half or more of the clones) seen in Patient M are indicated as follows; PNGS loss in yellow; gained in red; constant in green; PNGS composing the 2G12 epitope in blue. The exposed outer face as well as the V3 region of the gp120 monomer are indicated. The lost and gained PNGS are annotated in yellow and red, respectively. b) Side view of the gp120 trimer with the V3 region pointing downward toward the target cell.

Since end-stage R5 HIV-1 isolates analyzed in this study tended to be more sensitive to neutralization by the 2G12 MAb than by IgG1b12 or 2F5, we next compared 2G12 sensitivity with patient immune status, viral infectivity and Env characteristics. We established that R5 viruses neutralized by 2G12 (IC50 ≤25 µg/ml) were isolated from patients with reduced CD4^+^ T-cell count (p = 0.035; Figure 5a) and displayed increased infectivity when tested in U87.CD4-CCR5 cultures (p = 0.012; Figure 5b). In addition, the 2G12-sensitive viruses had Envs with reduced numbers of PNGS and increased positive net charge when compared to R5 isolates resistant to 2G12 neutralization (IC50 >25 µg/ml) (p = 0.015 and p = 0.018; Figures 5c and 5d). Since 2G12 binds to a cluster of high mannose glycans [37], [38] we mapped the localization of PNGS modifications in end-stage R5 viruses in relation to the 2G12 epitope. We observed that PNGS that were previously shown to be essential for 2G12 binding (N295, N332, N339, N386 and N392) were highly conserved in gp120 sequences from both 2G12 neutralization-sensitive and -resistant viruses (Table S1). Molecular models of gp120 were created based on Env sequences obtained before and after AIDS onset from patient M (Figure 6). The gp120 models comprised the core domain as well as the V3, V4 and V5 regions. Glycosylations were modelled at each PNGS as core saccharides composed of two *N*-acetylglycosamine and three mannose residues, found in all N-linked glycosylations. Comparative analysis of the molecular models suggested that glycans that were lost in R5 viruses following AIDS onset were predominantly localized to the outer solvent accessible domain of gp120 proximal to the 2G12 epitope (Figure 6). Furthermore the charge of the R5 isolates, calculated using the molecular models of the gp120 core (that lack mainly V1/V2), revealed increased positive charge in gp120 of 2G12-sensitive R5 isolates. This corresponded well to the net charge of gp160 derived from the amino acid sequence (data not shown). Increased positive surface charge was most prominent in the vicinity of the 2G12 epitope (Figure S2), when comparing molecular models of gp120 from chronic and end-stage R5 viruses from patient G, whose end-stage R5 virus also displayed the greatest gain in 2G12 sensitivity (Figure 5a). Thus, these results suggest that Env alterations, loss of PNGS and increase in surface positive charge, in the proximity to the 2G12 epitope, may play role in 2G12 neutralization.

## Discussion

In the present work, we demonstrate that R5 HIV-1 variants with increased sensitivity to the TriMAb mixture of broadly neutralizing IgGb12, 2F5 and 2G12 MAbs may emerge following AIDS onset at the end-stage of the disease. Increased sensitivity to TriMAb and to 2G12 alone coincided with enhanced viral infectivity and Env modifications, including reduced numbers of PNGS and increased positive charge. Such R5 virus variants appeared in patients with severe immunodeficiency, as evidenced by low CD4^+^ T-cell count at time of virus isolation.

Our data suggest that Env binding sites for neutralizing antibodies, such as the TriMAb mix, are better exposed in R5 viruses emerging *in vivo* in the absence of appropriate immune response, in a similar fashion to HIV-1 replicating in the newly infected host [24]. Indeed, in accordance with findings showing reduced glycan shield in Env of virus replicating during the acute infection [24], we and others [40], have found that Env of end-stage R5 virus displayed reduced glycosylation. It is noteworthy to mention that HIV-1 variants passaged extensively *in vitro* in the absence of anti-Env antibodies, i.e. T-cell line adapted viruses (TCLA), are known to be more sensitive to neutralizing antibodies as compared to primary isolates [44]–[46]. Thus, absence of immune pressure *in vivo* or *in vitro*, may result in reversion of escape or selection of minor virus variants being more sensitive to neutralizing antibodies, through a mechanism that includes loss of glycans.

Our findings also suggest that, in addition to Env glycan density, positive net charge of gp120 contributes to R5 virus sensitivity to neutralizing antibodies. Since sugar residues are negatively charged, reduction in glycan determinants may also contribute to elevation of gp120 surface charge. Increase in positive charge has been reported to have a positive effect on antibody-binding to the gp120 V3 region [47]. Our previous results disclosed gain in Env positive charge in other gp120 variable regions of R5 viruses at end-stage disease [18], suggesting that Env charge alterations outside the V3 loop also may influence virus sensitivity to neutralizing antibodies. Furthermore, end-stage R5 virus displayed increased *in vitro* fitness, assessed in primary cells and cell lines, as well as in competition assays [11], [17]. Interestingly, increase in viral infectivity of end-stage R5 viruses also coincides with reduction in Env PNGS numbers and increased sensitivity to neutralization by TriMab. In line with these results, Quekkelaar and colleagues showed that HIV-1 variants sensitive to either 2G12 or 2F5 neutralization displayed increased replicative capacity [48]. Recently it was also reported that broadly neutralizing antibodies frequently target a conserved epitope essential for viral fitness [49]. Thus, evolution of R5 HIV-1 in the absence of immune selection pressure at end-stage disease may, in an opportunistic manner, favour viral fitness rather than resistance to broadly neutralizing antibodies.

Increased sensitivity of end-stage R5 isolates to neutralization by the IgG1b12 Mab has been reported in previous studies where R5 isolates were obtained cross-sectionally before and after AIDS onset [12], [19]. In contrast, a longitudinal analysis of R5 isolates showed an increased resistance to IgG1b12 with disease progression [50]. We could not evaluate neutralization sensitivity using IgG1b12 and 2F5 since half of the analyzed R5 viruses were not neutralized to 50%. However, we found that 2G12-sensitive R5 isolates were obtained from patients with lower CD4^+^ T-cell counts than 2G12 resistant viruses. We also noted that increased 2G12 neutralization sensitivity correlated with reduced numbers of Env PNGS, which was surprising since the 2G12 epitope consists of specific oligomannose glycans positioned on the outer domain of gp120 [35], [37], [38]. Our Env sequence analysis revealed that the specific PNGS described to be important for 2G12 binding in large were conserved, indicating that the neutralizing effect of 2G12 may not solely be dependent on these specific glycans. Similarly, it has previously been reported that HIV-1 variants displaying 2G12 neutralization resistance may possess all critical PNGS [51], [52]. It has also recently been suggested that nonglycan determinants flanking the CD4 binding site influenced 2G12 neutralization via a mechanism involving shifts in the orientation of proximal glycans [53]. Thus, one potential explanation for our findings could be that the 2G12 epitope is more exposed if certain surrounding glycans are absent. Indeed, molecular modeling suggests that Env modifications, including PNGS loss and positive charge gain, acquired during end-stage disease development are located in the vicinity of glycans essential for 2G12 binding.

We believe that knowledge on the natural evolution of HIV-1 sensitivity to broadly neutralizing antibodies, linked to molecular alterations in the Env structure, may prove important for the understanding of mechanisms leading to virus escape and subsequent reversion from escape during HIV-1 progressive disease.

## Materials and Methods

### Patients and virus isolates

HIV-1 isolates were obtained from six patients selected from a larger cohort of homo- and bisexual men described previously [7]. This study was approved by the Karolinska Institute Regional Committee for Research Ethics, ref KI log no. 86:93. Oral consent was obtained from patients involved, in agreement with the decision of the Ethics Committee, and this was according to the ethical standards applied at the time when these isolates were obtained, between 1987–1995. The selected patients yielded R5 virus isolates throughout the entire course of the disease, including end-stage AIDS, and in the studied patients the R5 viral phenotype evolved by gain of enhanced fitness and reduced sensitivity to RANTES and entry inhibitors along with disease progression [10], [13], [17]. The samples were obtained before the advent of modern combination antiretroviral therapy (cART), but four of the patients (G, I, J and R) received monotherapy with zidovudine or didanosine. Isolations were made sequentially, at the chronic stage when the patients were clinically asymptomatic and after progression to AIDS at end-stage disease (Table 1). Primary virus isolates were previously obtained by isolation from peripheral blood mononuclear cells (PBMC) of infected individuals [7], as described [54]. Virus stocks were generated by propagation of isolates in PHA-stimulated (Boule) PBMC from healthy donors. The R5 phenotype was determined by infection of coreceptor indicator cell lines GHOST and U87 [10]. Isolates from patient R (6322 and 8004, see Table 1) displayed the ability to use both CCR5 and CCR3 in the indicator cell lines. However, since these isolates did not replicate in PBMC carrying the homozygous CCR5Δ32 genotype [10], they were classified as of R5 phenotype.

### HIV-1 neutralization assay

Virus neutralization sensitivity was analysed using the human MAbs IgG1b12, 2G12, 2F5 and equal molar ratio mixture of the three MAbs, known as TriMAb. All MAbs were either purchased from Polymun Scientific, Vienna, Austria or provided by NIH AIDS Research and Reference Reagent Program, Division of AIDS, NIAID, NIH. The neutralization assay was setup using the U87.CD4-CCR5 cell line as previously described [42], [43]. In brief,U87.CD4-CCR5 cells were maintained in Dulbecco's modified Eagle's medium (Invitrogen) supplemented with 10% fetal calf serum (Thermo Scientific) and antibiotics. One day prior to infection cells were seeded into 48-well plates in 500 µl of medium, to obtain a 50% confluent cell layer on the day of infection. At the day of infection, antibodies and virus stocks were diluted in infection medium, i.e. culture medium containing 2 µg/ml of Polybrene. The antibodies were diluted in four-fold steps, starting from a final concentration of 25 µg/ml present in the step when antibodies and virus were preincubated. Dilution of the virus stock was adjusted to results of a pretitration plaque test and a final inoculum of 40 plaque forming units/well was used. A mixture of 300 µl of diluted antibodies and 300 µl of diluted virus was preincubated in a separate 48-well plate for 1 h at 37°C. After the preincubation, the different antibody-virus mixtures were distributed into triplicate wells with U87.CD4-CCR5 in a volume of 200 µl well. Control cultures consisted of wells with cells and virus, but no antibodies. Two hours after infection 300 µl of infection medium was added to each well. After overnight incubation, cells were washed with PBS and 1 ml of infection medium was added to each well. Three days after infection the cells were washed with PBS and fixed with methanol-acetone (1∶1). To visualize cell nuclei, the fixed cells were stained with hematoxylin, washed with tap water and dried. The number of plaques (syncytia) was counted by light microscopy. The percent neutralization was calculated by determining the reduction in plaque forming units (p.f.u.)/well in the presence of inhibitory reagent compared with the control virus cultures containing no antibodies. The MAb concentrations resulting in 50% of plaque formation, IC50, was determined.

### U87.CD4-CCR5 infectivity assay

The infectivity assay has been described previously [17], [43] and resembles the neutralization assay described above. In brief, U87.CD4-CCR5 cells were seeded into 48-well plates and incubated over night to reach 50–60% confluence. Cells were infected with inoculum virus normalized to a concentration of functional viral reverse transcriptase (RT) of 8.5 ng RT ml^−1^ and then serially diluted in fivefold step. Functional RT was measured by the CAVIDI HS kit (Cavidi Tech AB, Uppsala, Sweden). On day five the cells were fixed and stained as stated above. The number of p.f.u. per ml was determined. Infectivity was tested for chronic and end-stage R5 viruses from five out of the six patients, where results from R5 viruses of patient J were lacking.

### Generation of full length env clones and sequence analysis

Full length *env* clones were generated from genomic DNA as described previously [11], [18]. Briefly, a 2.1 kb *env* fragment was amplified by nested PCR and cloned into the pSVIIIenv expression plasmid. The insert in the pSVIIIenv plasmid was used as template for sequence analysis of the *env* gene and from each R5 isolate four clones were selected according to functionality in a single round entry assay described previously [12], [55]. A set of 7 forward and 8 reverse primers and the ABI prism BigDye Terminator sequencing kit (Perkin Elmer) were used in the sequencing reaction. The sequenced segments were assembled to a contig sequence using the ContigExpress of VectorNTI Advance 10 software (Invitrogen). Sequences were aligned using ClustalX [56] followed by manual editing in GeneDoc [http://www.psc.edu/biomed/genedoc]. The obtained 48 *env* sequences were submitted to GenBank and assigned accession numbers [GenBank:EF600067-EF600114]. For determination of variation in potential N-linked glycosylation sites (PNGS) we used the N-glycosite tool in the HIV sequence database [http://www.hiv.lanl.gov] Maximum likelihood phylogenic trees showed patient as well as isolate-specific clustering, which argues against contamination and sample mix-up [18]. We defined the variable regions of gp120 as follows; V1 (nucleotide 6615–6692 in the HxB2 sequence), V2 (6693–6812), V3 loop (7110–7217), V4 loop (7377–7478) and V5 (7596–7637).

### Western blot

For analysis of Env expression and proteolytic cleavage, 293T cells were cotransfected with pSVIIIenv plasmid and pSVTat plasmids at an 8∶1 ratio using Lipofectamine 2000. At 72 hrs after transfection, cells were resuspended in ice cold lysis buffer (0.5% [vol/vol] NP-40, 0.5% [wt/vol] sodium deoxycholate, 50 mM NaCl, 25 mM Tris-HCl [pH 8.0], 10 mM EDTA, 5 mM benzamidine HCl), and a cocktail of protease inhibitors (Roche) for 10 min, followed by centrifugation at 13000× *g* for 10 min to remove cellular debris. Cell lysates were separated in 8.5% (wt/vol) sodium dodecyl sulfate-polyacrylamide gel electrophoresis (SDS-PAGE) and Env proteins were detected by Western blotting using rabbit anti-gp120 polyclonal antisera. Env proteins were visualized using horseradish peroxidase-conjugated antirabbit immunoglobulin G antibody and enhanced chemi-luminescence (Promega). To investigate the extent of glycosylation, cell lysates were incubated with PNGase F (Sigma) according to the manufacturer's protocol. Briefly, cleared cell lysate was incubated over night with 50000 U/ml PNGase (Sigma), at 37°C prior to SDS-PAGE and Western blotting.

### Molecular modelling of gp120 from chronic- and end-stage R5 viruses

A molecular model of the monomeric HIV-1 gp120 was generated based on the crystal structure of the HIV-1 gp120 (PDB ID 2B4C) using the SWISS-MODEL protein modeling server [57]. The program Coot [58] was further used for minor localized adjustments. The trimeric model of HIV-1 gp120 was created using the recently published model of the CD4-bound gp120 trimer derived from three-dimensional cryo electron tomography studies [59]. Glycans were modelled at each PNGS, using the GLYPROT glycan modelling server (http://www.glycosciences.de/modeling/glyprot/php/main.php), as pentasaccharide structures common to all N-linked sites composed of two *N*-acetylglycosamine and three mannose residues [GlcNAc-GlcNAc-Man-(Man)_2_]. The 2G12 epitope was defined for the modelling as composed of PNGS 295, 332, 339, 386 and 392 [37], [38]. Total charges of the molecular models prior to glycan addition were calculated using the PROPKA server (version 3.0 http://propka.ki.ku.dk/) with standard settings at pH 7.0 [60], [61]. Vacuum electrostatic surface potentials were visualized through calculation of locally averaged surface charges using the protein contact potential visualization as implemented in Pymol. All figures were prepared using Pymol (PyMOL Molecular Graphics System, Version 1.2r1, Schrödinger, LLC).

### Statistical analysis

For statistical analysis we used the Statistica software version 7. The non-parametric Spearman rank correlation was used for the analysis of correlations. Comparisons between chronic and end-stage Env sequences and antibody sensitivity were conducted with Wilcoxon's matched pairs test. Non-parametric Mann-Whitney U-test was used when comparing 2G12 sensitivity to viral and clinical features.

## Supporting Information

Figure S1 **Localization of PNGS modifications in gp120 of HIV-1 R5 emerging during end-stage disease.** Localization of PNGS in gp120 of chronic and end-stage R5 virus, calculated from four sequenced clones per R5 isolate. The percentage of clones with a PNGS at a given position is color coded with increasingly darker shades of gray.
(PDF)

Figure S2 **Changes in electrostatic surface potential in a molecular model of the gp120 trimer comparing chronic and end-stage R5 viruses** Visualisation of the electrostatic surface potential of molecular models of trimeric gp120 from chronic and end-stage R5 virus of patient G. Positively and negatively charged parts are shown in blue and red, respectively. The gp120 trimer is presented from the side with the V3 region pointing downward toward the target cell, and the approximate location of the 2G12 core epitope is depicted with arrows.
(TIF)

Table S1 **2G12 epitope in sequential R5 Env sequences and 2G12 IC50 for corresponding R5 isolates.**
(PDF)
