# Supplementary figures and images for: Increased Sensitivity to Broadly Neutralizing Antibodies of End-Stage Disease R5 HIV-1 Correlates with Evolution in Env Glycosylation and Charge
Source: PLoS One. 2011 Jun 16;6(6):e20135. doi: 10.1371/journal.pone.0020135 (PMC3116816; doi:10.1371/journal.pone.0020135)

Figure S1.

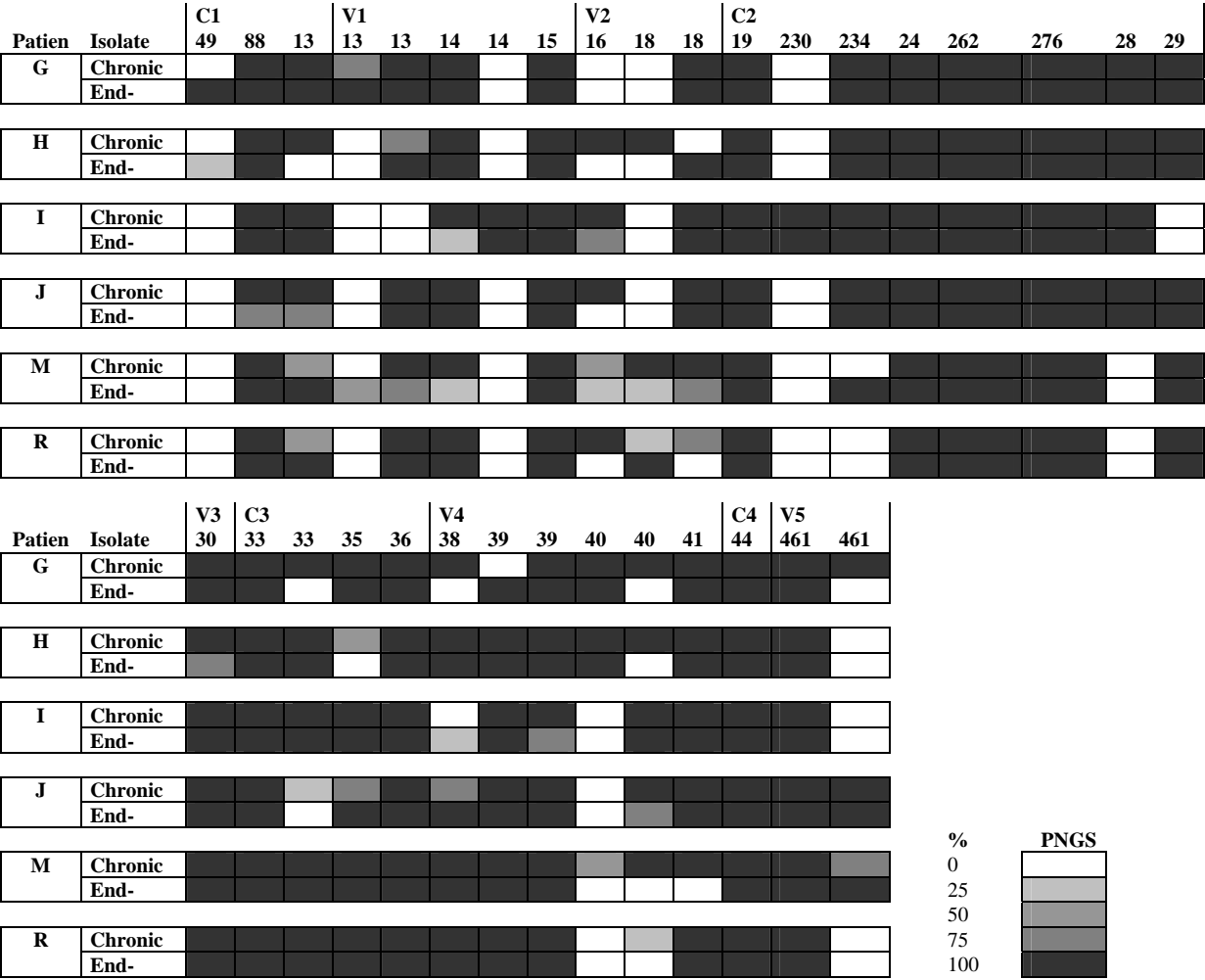

Supplement: Figure S1 — Localization of PNGS modifications in gp120 of HIV-1 R5 emerging during end-stage disease. Localization of PNGS in gp120 of chronic and end-stage R5 virus, calculated from four sequenced clones per R5 isolate. The percentage of clones with a PNGS at a given position is color coded with increasingly darker shades of gray. (PDF) [file pone.0020135.s001.pdf]

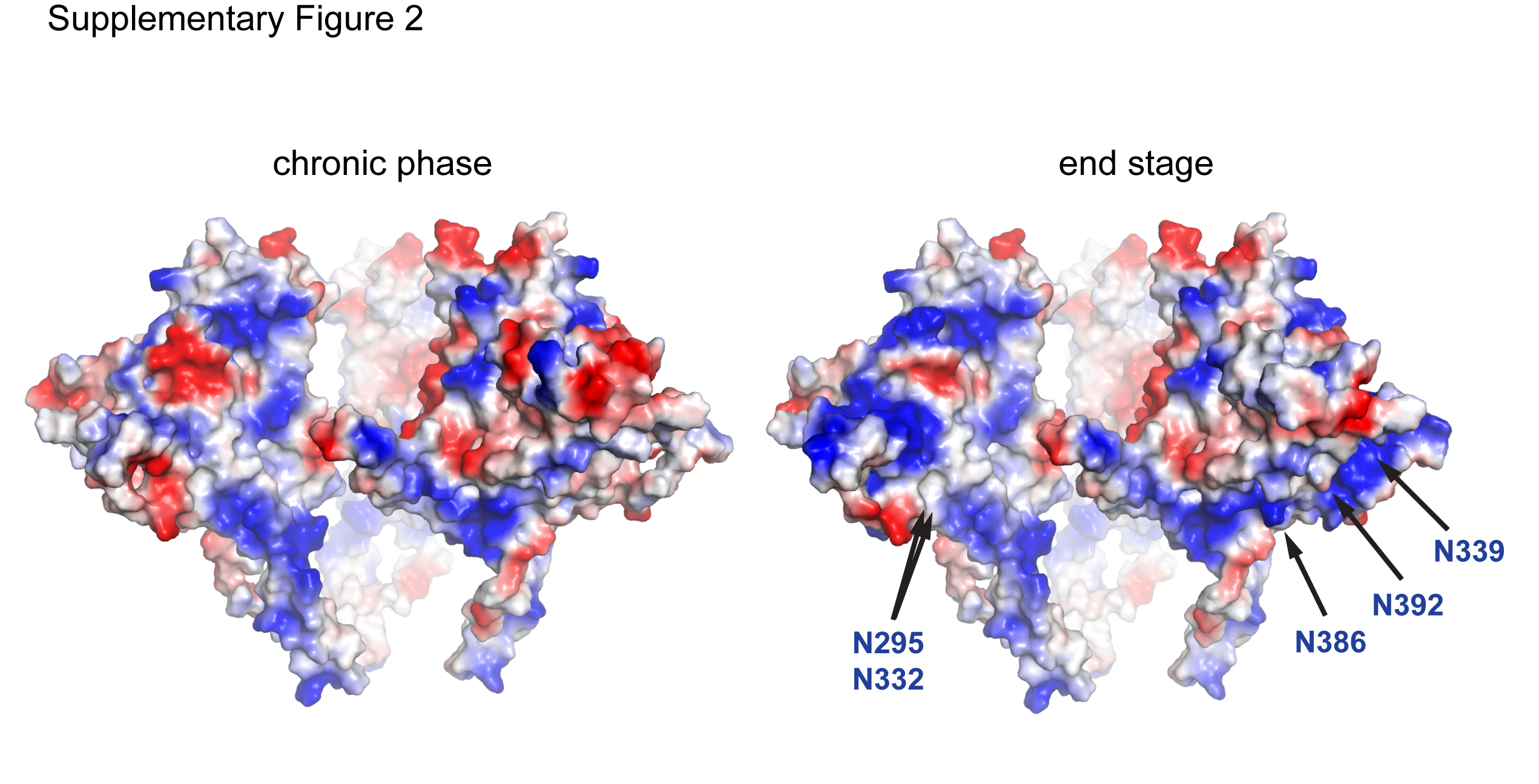

Supplement: Figure S2 — Changes in electrostatic surface potential in a molecular model of the gp120 trimer comparing chronic and end-stage R5 viruses Visualisation of the electrostatic surface potential of molecular models of trimeric gp120 from chronic and end-stage R5 virus of patient G. Positively and negatively charged parts are shown in blue and red, respectively. The gp120 trimer is presented from the side with the V3 region pointing downward toward the target cell, and the approximate location of the 2G12 core epitope is depicted with arrows. (TIF) [file pone.0020135.s002.tif]
