# Supplementary material for: Increased Sensitivity to Broadly Neutralizing Antibodies of End-Stage Disease R5 HIV-1 Correlates with Evolution in Env Glycosylation and Charge
Source: PLoS One. 2011 Jun 16;6(6):e20135. doi: 10.1371/journal.pone.0020135 (PMC3116816; doi:10.1371/journal.pone.0020135)
Supplement: Table S1 — 2G12 epitope in sequential R5 Env sequences and 2G12 IC50 for corresponding R5 isolates. (PDF) [file pone.0020135.s003.pdf]

Table S1. 2G12 epitope in sequential R5 Env sequences and 2G12 IC50 for corresponding R5 isolates

			2G12 epitope[b]					
**Patient[a]**	**Isolate**	**Clinical status**	295	332	339	386	392	**2G12 IC50 ug/ml**
G	1228	Chronic	+	+	+	+	-	>25
	4481	End-stage	+	+	-	-	+	3,95
H	624	Chronic	+	+	+	+	+	>25
	3899	End-stage	+	+	+	+	+	20,16
I	5013	Chronic	-	+	+	-	+	>25
	8616	End-stage	-	+	+	- (+ 1/4[c])	+	>25
J	1372	Chronic	+	+	- (+ 1/4[c])	+ (- 1/4[d])	+	4,87
	5714	End-stage	+	+	-	+	+	14,24
M	668	Chronic	+	+	+	+	+	>25
	7363	End-stage	+	+	+	+	+	13,47
R	6322	Chronic	+	+	+	+	+	1,16
	8004	End-stage	+	+	+	+	+	0,36

a) Patient code according to [10]

b) Data based on sequences of four clones; + presence of PNGS in 4/4 clones, - absence of PNGS in 4/4 clones.

c) The sequence of one clone out of four had the PNGS

d) The sequence of one clone out of four lacked the PNGS
